# Smartphone Medical App Use and Associated Factors Among Physicians at Referral Hospitals in Amhara Region, North Ethiopia, in 2019: Cross-sectional Study

**DOI:** 10.2196/19310

**Published:** 2021-03-26

**Authors:** Gizaw Hailiye Teferi, Binyam Cheklu Tilahun, Habtamu Alganeh Guadie, Ashenafi Tazebew Amare

**Affiliations:** 1 Department of Health Informatics College of Medicine and Health Science Debre-Markos University Debre Markos Ethiopia; 2 Department of Health Informatics College of Medicine and Health Sciences University of Gondar Gondar Ethiopia; 3 School of Public Health College of Medicine and Health Science Bahir Dar University Bahir Dar Ethiopia; 4 Department of Pediatrics College of Medicine and Health Science University of Gondar Gondar Ethiopia

**Keywords:** application, medical, physician, smartphone, mobile phone

## Abstract

**Background:**

Information in health care is rapidly expanding and is updated very regularly, especially with the increasing use of technology in the sector. Due to this, health care providers require timely access to the latest scientific evidence anywhere. Smartphone medical apps are tools to access the latest reputable scientific evidence in the discipline. In addition, smartphone medical apps could lead to improved decision making, reduced numbers of medical errors, and improved communication between hospital medical staff.

**Objective:**

The aim of this study was to assess smartphone medical app use and associated factors among physicians working at referral hospitals of the Amhara region, Ethiopia.

**Methods:**

An institution-based cross-sectional study design was conducted among physicians working at 5 referral hospitals in the Amhara region, Ethiopia, from February 5 to May 27, 2019. A simple random sampling method was used to select 423 physicians. A self-administered questionnaire was used to collect the data and analyzed using SPSS, version 21 (IBM Corp). Binary and multivariable logistic regression analysis was performed to assess factors associated with smartphone medical app use among physicians. A value of *P*<.05, corresponding to a 95% CI, was considered statistically significant. The validity of the questionnaire was determined based on the view of experts and the reliability of it obtained by calculating the value of Cronbach alpha (α=.78)

**Results:**

In this study, most of the 417 respondents (375, 89.9%) had medical apps installed on their smartphones. Of those 375 respondents, 264 (70.4%) had used medical apps during clinical practice. The medical apps most commonly used by the respondents were UpToDate, Medscape, MedCalc, and Doximity. According to multivariable logistic regression analysis, attitude (adjusted odds ratio [AOR] 1.64, 95% CI 1.05-2.55), internet access (AOR 2.82, 95% CI 1.75-4.54), computer training (AOR 1.71, 95% CI 1.09-2.67), perceived usefulness of the app (AOR 1.64, 95% CI 1.05-2.54), information technology support staff (AOR 2.363, 95% CI 1.5-3.08), and technical skill (AOR 2.52, 95% CI 1.50-4.25) were significantly associated with smartphone medical app use.

**Conclusions:**

Most respondents have a smartphone medical app and have used it in clinical practice. Attitude, internet access, computer training, perceived usefulness of the app, information technology support staff, and technical skill are the most notable factors that are associated with smartphone medical app use by physicians.

## Introduction

The main sources of information for health care professionals at the point of care were once textbooks [1]. However, health care professionals increasingly use smartphone medical apps for patient care, clinical reference, and education [2]. The smartphone is a tool that has recently grown in use and has been accepted by health professionals and medical students. It is a new technology that has an operating system, the capability of installing various apps, and the ability to do complex calculations and establish related communications at the point of care [1,3]. Smartphone apps are tools that can be downloaded onto smartphones or computer tablets and enhance patient care, increase efficiency, or provide individualized learning for clinicians [4]. In 2011, Apple created the Apps for Healthcare Professionals section within the medical category of the iTunes App Store, a unique feature among mobile app marketplaces [5]. Smartphones have a wide range of uses from the internet to email; they offer on-the-go access to information that was never before possible [6]. The most commonly used smartphone medical apps are UpToDate and Medscape, as shown in Table 1.

**Table 1:** Summary of the most commonly used smartphone medical apps.

Rank	App	App category	Operating system
1	UpToDate	Medical reference	iOS, Android, Windows
2	Medscape	Medical reference	iOS, Android, Windows
3	Epocrates	Drug and medical reference	iOS, Android, Windows
4	PEPID	Decision support/reference	iOS, Android, Windows
5	Figure 1	Medical image	iOS, Android, Windows
6	MedCalc	Drug reference	iOS, Android, Windows
7	Prognosis	Decision support/reference	iOS, Android, Windows
8	Skyscape	Drug and medical reference	iOS, Android, Windows
9	Diseases Dictionary Medical	Medical disorders & diseases with detailed definitions, symptoms, causes, and treatment information	iOS, Android, Windows
10	Calculate by QxMD	Literature and drug reference	iOS, Android, Windows

A study conducted in the United Kingdom showed that smartphone medical apps like British National Formulary, eLogbook, and medical calculator (MedCalc) have been commonly used by physicians [7]. This technology can lead to improved decision making, reduced numbers of medical errors, and improved communication between hospital medical staff [8-10].

Another study from the United Kingdom stated that due to the ease of use of smartphone medical apps, 18.5% of doctors made suggestions to their colleagues to use apps as a quick reference during clinical practice [7]. However, lack of support and updating of apps by their developers, lack of adequate skill to use apps, lack of creating motivation in using apps, and problems related to security and confidentiality of patient information have undermined the use of smartphone medical apps at the point of care [10-12]. A study conducted in Korea showed low use of smartphone medical apps by physicians [13].

Evidence shows that medical app use is high in high-income countries; compared with the Korean study, a study conducted in the United Kingdom found higher medical app use (72.4%) among doctors [14]. 

Another study in the United States reported that 56% of physicians use apps in their clinical practice. There was a decreasing trend in app use with increased training level, and the most useful app types included textbook and reference materials (average response: 55%), classification and treatment algorithms (46%), and general medical knowledge (43%); there was a greater desire for apps among residents than among fellows and attending physicians [15]. This might be due to residents being less experienced physicians than the senior specialists and subspecialists; as a result, they need some assistance from colleagues and seniors, so medical apps are immediate reference tools that can be accessed anywhere during clinical practice. This explains why the level of use among residents is higher than among their senior attending physicians.

A study conducted in Saudi Arabia showed the use patterns of smartphone medical apps among residents at clinical practice for counselling and clinical communication (50%), among interns for drug reference (56%), and among externs for resources and e-books (65%) [16].On the other hand, a study done in Iran reported that the most popular medical apps were Medscape and UpToDate, and 61.3% of the physicians were using their apps more than once a day, mostly for drug information [17].

According to a cross-sectional study done in Ghana, over 43.1% of physicians frequently used medical apps on their smartphones for clinical decision making, which shows relatively low use of medical apps compared with that in high-income countries [18].

Due to different factors, physicians remain reluctant to adopt these technologies in clinical practice [2]; the most common factors that affect the use of smartphone medical apps are behavioral factors (information technology (IT)–related experience, attitude, computer-related skill) [19], factors related to medical app characteristics (perceived usefulness, perceived ease of use, privacy and security concerns), organizational factors (infrastructure, IT support, and computer-related training) [20]. Shreds of evidence revealed that underutilization of apps in clinical practice by health care professionals is due to a lack of technical skill [21]. According to the findings of a cross-sectional study conducted in the United Kingdom, appearing to be looking at a phone during clinical practice could be misinterpreted as checking emails or using social networks by colleagues and patients [7,22-25].

Security and privacy are the key factors of the functionality of any mHealth system [26]; unfortunately, most of the time these important areas are neglected by development teams of mHealth systems, and the majority of currently available mHealth apps impart little or no security. This will affect the use of these apps significantly [27-29]. Perceived ease of use is another factor that determines the use of smartphone medical apps; those with a user-friendly interface are more likely to be used [23]. The aim of this study was to assess the level of smartphone medical app use and associated factors among physicians in referral hospitals of the Amhara region, Ethiopia.

## Methods

### Study Design and Setting

This was a cross-sectional, questionnaire-based study done to assess smartphone medical app use and associated factors among physicians in referral hospitals (Gondar University, Felege Hiwot, Debre Markos, Dessie, and Debre Birhan referral hospitals) in Amhara region, Ethiopia, which consists of 10 administrative zones, 1 special zone, 181 woredas, and 78 urban centers. The capital city of the state of Amhara is Bahir-Dar [30]. It is located in the northwestern and north-central parts of Ethiopia. The state shares common borders with the state of Tigray in the north, Afar in the east, Oromia in the south, Benishangul/Gumuz in the southwest, and the Republic of Sudan in the west [31].

The sample size was computed as 423, which was 80% of the total population during the data collection period, using a single population proportion formula taking 50% at a 95% confidence level and assuming a 5% margin of error and 10% nonresponse rate. There are 5 referral hospitals in the region; all 5 referral hospitals were included, and then proportional allocation was made for each hospital. Finally, a simple random sampling method was used. 213 permanent doctors (general practitioners and specialists), 95 residents, and 115 interns were recruited using a lottery method (each member of the population was assigned a unique number, each number was written on a separate piece of paper or card of the same size, the cards were mixed well in a basket, and the sample was drawn) and formed the sample.

The survey consisted of 36 questions encompassing the following domains: (1) sociodemographic characteristics, (2) attitude, (3) factors related to medical app characteristics (perceived usefulness and perceived ease of use of apps), (4) physician technical skill, and (5) organizational factors [20].

Attitude was assessed using a Likert scale (1=strongly disagree, 2=disagree, 3=neutral, 4=agree, 5=strongly agree) for the following questions: Do you believe smartphone medical apps give you greater control over your work schedule? Do you believe smartphone medical apps will improve your work performance? Do you think smartphone medical apps allow you to conduct your job more quickly?

Organizational factors were assessed with yes/no questions as follows: Do you have internet access in your office at clinical practice? Have you ever taken any training on use of smartphone medical apps? Does your organization have IT support staff?

Perceived usefulness and ease of use of medical apps were assessed using a Likert scale (1=strongly disagree, 2=disagree, 3=neutral, 4=agree, 5=strongly agree) for questions such as the following: Do you think learning to use smartphone medical apps will not require much time? Do you think smartphone medical apps are easy to use? Do you think smart phone medical apps are easy to use during your consultations with patients?

Smartphone medical app use was assessed using 9 categories of medical apps: (1) drug reference, (2) clinical score systems, (3) disease diagnosis, (4) procedure documentation, (5) literature search, (6) clinical communication, (7) health information system clients, (8) medical training, and (9) web browsing. Physicians who used 5 or more of the app categories, which was the median, were categorized as having used smartphone medical apps and those who used fewer than 5 were not.

A self-administered questionnaire was adopted from previous studies [14,32]. To ensure the validity of the questionnaire, an expert panel (10 doctors having at least 5 years’ experience in general practice or primary care research) was invited to review the tool and revise it, and reliability was calculated to be α=.78.

Before the actual data collection, pilot testing of the questionnaire was conducted among 20 physicians at Debre Tabor hospital to check internal consistency within the questioners. Then necessary correction was done based on the pretest finding.

Two days’ training was given for 5 data collectors on the objective of the study and data collection procedures. Data was collected from January 15 to March 30, 2019, using self-administered questionnaires; one data collector was assigned for each hospital, and the supervisor facilitated the data collection process. The principal investigator and supervisors did daily supportive supervision on data collectors. Data backup activities, such as storing data at different places and putting data in different formats (hard and soft copies), were performed to prevent data loss.

### Study Variables and Operational Definitions

#### Dependent Variable

The dependent variable was the physician’s smartphone medical app use.

#### Independent Variables

The independent variables were sociodemographic factors (age, sex, profession, educational status, experience), medical app–related factors (perceived usefulness, perceived ease of use, privacy and security concerns), organizational factors (internet access, IT support, computer-related training), and behavioral factors (knowledge, attitude, technical skill, IT-related experience).

#### Operational Definitions

In this study, “physician” includes general practitioners, residents, dentists, specialists, and subspecialists.

Smartphone is a class of mobile phone with multipurpose mobile computing capability and features like high-definition camera, third-party app installation, and global positioning system [33].

Medical apps are computer programs or software apps that are designed to run on a mobile device such as a smartphone or tablet and are meant to be used for clinical purposes.

Smartphone medical apps are medical apps designed to run specifically on smartphones [34].

Study participants who scored at or above the median of 5 out of the 9 categories of Food and Drug Administration–approved medical apps were categorized as having used smartphone medical apps [18].

For the attitude questions, study participants who scored the median or higher on the 5-point Likert scale were categorized as having a good attitude, and those who scored below the median were categorized as having a poor attitude [35].

For the perceived usefulness questions, study participants who scored the median or higher on the 5-point Likert scale were categorized as thinking smartphone medical apps were useful for their job, and those who scored below the median were categorized as thinking smartphone medical apps were not useful for their job [36].

For the perceived ease of use questions, study participants who scored the median or higher on the 5-point Likert scale were categorized as thinking smartphone medical apps were easy to use, and those who scored below the median were categorized as thinking smartphone medical apps were not easy to use.

### Data Processing and Analysis

Data were entered into Epi Info, version 7 (Centers for Disease Control and Prevention), and exported to SPSS, version 21 (IBM Corp), for further analysis. Descriptive statistics were computed to summarize variables, and the binary logistic regression model was used to measure the association between dependent and independent variables. Both crude odds ratios for binary logistic regression analysis and adjusted odds ratios (AOR) for multivariable logistic regression analysis were estimated with 95% CIs to show the strength of associations. Finally, a *P* value of less than .05 in the multivariable logistic regression analysis was used to identify variables significantly associated with the use of smartphone medical apps.

### Ethical Considerations

In conducting the study, ethical clearance was obtained from the University of Gondar ethical review board. Additional permissions to access participants were also obtained from each hospital administrator. In addition, written informed consent was gained from all participants (Multimedia Appendix 1), participation in the study was voluntary, and no incentive was provided for the participants.

## Results

### Sociodemographic Characteristics

A total of 417 physicians were included in this study with a response rate of 98.6% (417/423). Two-thirds (275, 65.9%) of the respondents were male. The mean age was 33 years (SD 8) with the majority in the age group of 25-34 years. More than three-fourths (375, 89.9%) of the physicians had the medical app installed on their smartphones (Table 2).

**Table 2:** Sociodemographic characteristics of respondents working at referral hospitals of Amhara region, North Ethiopia, 2019 (N=417).

Variable			n (%)
**Gender**			
	Male	275 (65.9)	
	Female	142 (34.1)	
**Age (years)**			
	≤30	217 (52.0)	
	>30	200 (48.0)	
**Educational status**			
	General practitioner	219 (52.5)	
	Resident	127 (30.5)	
	Specialist	71 (17.0)	
**Department**			
	Internal medicine	80 (19.2)	
	Pediatrics	54 (12.9)	
	Radiology	28 (6.7)	
	Surgery	67 (16.1)	
	Ophthalmology	25 (6.0)	
	Gynecologist	65 (15.6)	
	Dermatology	18 (4.3)	
	ENT^a^	36 (8.6)	
	Other	44 (10.6)	
**Work experience (years)**			
	1-3	231 (55.4)	
	3-6	91 (21.8)	
	>6	95 (22.8)	
**Medical app ownership**			
	Yes	375 (89.9)	
	No	42 (10.1)	

^a^ENT: ear, nose, and throat.

### Smartphone Medical App Use of Physicians at Referral Hospitals

According to this study, 63.3% (264) of the respondents reported that they use apps in their clinical practice (95% CI 58.3%-67.9%), and the most commonly used smartphone medical app category was diagnosis/management (62%) (Table 3).

According to this study, the most commonly used smartphone app was UpToDate (300/417, 71.9%) (Table 4).

Most study respondents (354, 85%) used their apps daily, while 10.5% used them 3 times a week (Table 5).

**Table 3:** Smartphone medical app use at referral hospitals among physicians, 2019 (N=417).

Medical app types	Education level			
	General practitioner, n	Resident, n	Specialist, n	Total, n (%)
Disease diagnosis	134	84	43	261 (62.6%)
Literature search	120	67	31	218 (52.3%)
Browsing	113	54	29	196 (47.0%)
HIS^a^ clients	106	54	35	195 (46.8%)
Clinical score system	104	71	34	209 (50.1%)
Medical training	104	59	31	194 (46.5)
Drug reference	97	48	28	173 (41.5)
Clinical communication	87	58	23	168 (40.3%)
Procedure documentation	73	51	28	152 (36.5%)

^a^HIS: health information system.

**Table 4:** Medical apps used by physicians working at referral hospitals in Amhara regional state, Ethiopia, 2019.

App used		n (%)
**UpToDate**		
	Yes	305 (73.1)
	No	112 (26.9)
**Medscape**		
	Yes	276 (66.2)
	No	141 (33.8)
**MedCalc**		
	Yes	226 (54.2)
	No	192 (46.0)
**Doximity**		
	Yes	104 (24.9)
	No	313 (75.1)
**PEPID**		
	Yes	50 (12.0)
	No	367 (88.0)
**Case**		
	Yes	97 (23.3)
	No	320 (76.7)
**Figure 1**		
	Yes	99 (23.7)
	No	318 (76.3)
**Read by QxMD**		
	Yes	90 (21.6)
	No	327 (78.4)

**Table 5:** Frequency of smartphone medical app use among physicians working at referral hospitals of Amhara regional state, 2019 (N=375).

Medical app	Frequency of use, n (%)			
	Daily	Three times a week	Once a week	I don’t know
UpToDate	333 (88.8)	28 (7.5)	12 (3.2)	2 (0.5)
Medscape	335 (89.3)	25 (6.7)	13 (3.5)	2 (0.5)
MedCalc	346 (92.3)	18 (4.8)	9 (2.4)	2 (0.5)
Doximity	340 (90.7)	23 (6.1)	12 (3.2)	0
Figure 1	339 (90.4)	24 (6.4)	12 (3.2)	0
Read by QxMD	335 (89.3)	27 (7.2)	13 (3.5)	0
Case	346 (92.3)	21 (5.6)	8 (2.1)	0
PEPID	322 (85.9)	18 (4.8)	35 (9.3)	0

### Factors Associated With Smartphone Medical App Use Among Physicians

A total of 6 variables were selected as potential predictors of smartphone app use after bivariable logistic regression and entered multivariable logistic regression. Included variables were attitude, internet access, computer training, past IT experience, perceived ease of use of the app, perceived usefulness of the app, the technical skill of the physicians, and availability of IT support staff, which were positively related to smartphone medical app use by physicians at referral hospitals in Amhara region.

In this study, physicians with a favorable attitude toward smartphone medical apps were 1.64 times more likely to use them than physicians with an unfavorable attitude were (AOR 1.64, 95% CI 1.05-2.55). Similarly, physicians who have IT support staff at hospitals were 2.36 times more likely to be smartphone medical app users compared to their counterparts (AOR 2.36, 95% CI 1.5-3.08) (Table 6).

**Table 6:** Bivariable and multivariable regression analysis of factors with smartphone medical app use among physicians in referral hospitals of Amhara regional state, North Ethiopia, 2019 (N=417).

Variable		App use, n (%)		Crude OR^a^ (95% CI)	AOR^b^ (95% CI)	*P* value
		Yes	No			
**Education level**						
	GP^c^	142 (34.1)	77 (18.5)	1.51 (0.87-2.60)	1.68 (0.91-3.10)	—^d^
	Resident	83 (19.9)	44 (10.6)	1.54 (0.85-2.80)	1.70 (0.88-3.30)	—
	Specialist	39 (9.4)	32 (7.7)	1	1	—
**Internet access**						
	Yes	210 (50.4)	88 (21.1)	2.87 (1.85-4.45)	2.82 (1.75-4.50)	<.001
	No	54 (12.9)	65 (15.6)	1	1	—
**Computer training**						
	Yes	179 (42.9)	72 (17.3)	2.36 (1.57-3.56)	1.71 (1.09-2.67)	<.001
	No	85 (20.4)	81 (19.4)	1	1	—
**IT support staff**						
	Yes	156 (37.4)	58 (13.9)	2.366 (1.57-3.56)	2.363 (1.50-3.08)	.001
	No	108 (25.9)	95 (22.8)	1	1	—
**Technical skill**						
	Yes	225 (54)	103 (24.7)	2.8 (1.73-4.52)	2.54 (1.50-4.30)	<.001
	No	39 (9.4)	50 (12.0)	1	1	—
**Attitude**						
	Yes	153 (36.7)	69 (16.5)	1.67 (1.12-2.50)	1.64 (1.05-2.55)	.01
	No	111 (26.6)	84 (20.1)	1	1	—
**Perceived usefulness**						
	Yes	158 (37.9)	70 (16.8)	1.76 (1.18-2.64)	1.65 (1.06-2.56)	.02
	No	106 (25.4)	83 (19.9)	1	1	—

^a^OR: odds ratio.

^b^AOR: adjusted odds ratio.

^c^GP: general practitioner.

^d^Not available.

## Discussion

### Principal Findings

This study assessed the use of smartphone medical apps and associated factors among physicians at referral hospitals in the Amhara region. Out of 417 participants, 375 (89.9%) have a medical app installed on their mobile device, and disease diagnosis/management was the most commonly used medical app category. Factors like attitude, perceived usefulness, internet access, and past computer-related training were found to be associated with smartphone medical app use. In this study, the use rate of smartphone medical apps by physicians was 63.3% (95% CI 58.3%-67.9%). This result was consistent with that of a study done in Britain (60%) [6]. On the other hand, it was lower than those of a study done in Canada (77.0%) [11], a study conducted in Germany at the Leipzig Medical School (68%) [37], and a study conducted in the United States on the American Society of Plastic Surgeons (72%) [38]. This might be due to well-organized infrastructure at the clinical practice site, awareness of physicians on the use of smartphone medical apps for patient care, and availability of technological guidelines that promote the use of smartphone medical apps in America, Canada, and Germany. On the other hand, the result is higher than that of a study in Ghana (43.1%) [18]. A possible explanation may be due to the sample size difference (the sample size of the study in Ghana was 65) and the study period (this study was conducted about 4 years ago).

 This study indicated that the medical apps most frequently used by physicians were UpToDate, Medscape, and MedCalc. This result is in line with the study conducted in Canada [11]. Most of the physicians (271/375, 72.3%) preferred smartphone medical apps as an information source for reference during clinical practice. The proportion is lower in the study in Ghana than in this study; this might be due to the accessibility of smartphones in our setup and sample size difference (the sample size of the study in Ghana was 65) [18]. 

This study found that the perceived usefulness of smartphone medical apps was positively associated with medical app use among physicians at referral hospitals in the Amhara region (*P*=.04). This is in line with a study conducted in Taiwan [39]. Perceived usefulness of apps was a significant determinant of app use according to a study conducted in a Malaysian public hospital [40]. That result is comparable with this current study (OR 1.65, 95% CI 1.06-2.56]). 

This study revealed that physicians who had good technical skills were 2.54 times more likely to use smartphone medical apps at clinical practice than those who had poor technical skills (AOR 2.54, 95% CI 1.50-4.30]). A study conducted in Czech Republic also indicated that technical skill was a factor for smartphone app use [41]. This might be because people with good technical skills are more receptive to new technology and capable of operating new apps. 

According to this study, physicians who were working in an institution with internet access (WiFi) were 2.82 times more likely to use smartphone medical apps than those who had no internet access (AOR 2.82, 95% CI 1.75-4.54). This might be because the availability of internet access makes the regular update of medical apps easier and makes it possible to exchange information through a medical app, such as for consultation among senior physicians. 

Physicians who were in an institution that has IT support staff were 2.36 times more likely to use smartphone medical apps than their counterparts (AOR 2.363, 95% CI 1.50-3.08). 

This study revealed that the odds of physicians with favorable attitudes being users of smartphone medical apps were 1.64 times higher than those of their counterparts (AOR 1.64, 95% CI 1.05-2.55), which is in line with the result obtained by a study conducted in Iran [35]. 

This implies that the attitude of physicians is key in the implementation of such apps in clinical practice. From the results above, we found that smartphone medical apps used by physicians did well in providing relevant medical information during clinical practice and received positive reviews from physicians. However, in other aspects (ie, outside of improving clinical decision making, saving time, helping to make differential diagnoses, performing useful medical-related calculations, and providing faster access to evidence-based medical practices or cases), medical apps did not meet the needs of physicians well, as most of the medical apps are not freely accessible, the cost of these apps is not affordable, and more importantly, the payment mechanism is not available in our country, Ethiopia. Therefore, in the future, there will be much room for improvement, and health care institutions in resource-limited countries like Ethiopia should offer an institutional access mechanism to such medical apps that is accessible freely.

### Conclusion

The findings of this study showed that smartphone medical app use was 63.3%. Favorable attitude, internet access, computer training, perceived usefulness of the app, the technical skill of the physicians, and availability of IT support staff were the most notable factors that were associated with smartphone medical app use.

Based on this result, smartphone medical apps have inevitable contributions to successful and effective clinical practice. To effectively use this technology in clinical practice, health care organizations should create awareness of its use and implications in health care service, improve internet connectivity, and provide training on the use of these apps.

## Appendix

Multimedia Appendix 1 Informed consent statement.

## Abbreviations

AOR: adjusted odds ratio
IT: information technology
mHealth: mobile health
