# Supplementary material for: Smartphone Medical App Use and Associated Factors Among Physicians at Referral Hospitals in Amhara Region, North Ethiopia, in 2019: Cross-sectional Study
Source: JMIR Mhealth Uhealth. 2021 Mar 26;9(3):e19310. doi: 10.2196/19310 (PMC8096376; doi:10.2196/19310)
Supplement: Multimedia Appendix 1 [file mhealth_v9i3e19310_app1.docx]

**Informed consent statement**

**University of Gondar College of Medicine and Health Sciences**

**Institute of Public Health**

**Questionnaire for assessment Smartphone Medical application use among Physicians and associated factors at referral hospitals in Amhara regional state, Ethiopia 2019**

Hello! My name is __________________________I am here on behalf of Gizaw Hailiye, student of Master of Public Health in Health informatics at University of Gondar College of Medicine and Health Sciences, Institute of Public Health. He has ethical clearance from University of Gondar to conduct the research on the above topic. You are chosen to participate in this study and your anonymous answers will be used only for research purpose. In order to effectively attain the purpose of the research I request you to give genuine response to each question. There are questions for you to complete and there is no need to put your name on the questionnaire; no individual responses will be reported. Your answers are completely confidential. It is your full right to refuse, to answer any or all of the questions. If you don't want to participate you can leave the questionnaire empty. Study questionnaires will take maximum of 20 minutes.

**Certificate of consent**

I understand that the findings of this research will be disseminated to Hospital management and decision makers that will be useful as an input for intervention design.

I voluntarily consent to participate in this study.

I agree I disagree


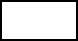


If you are agree to participate in the study please visit the next page.

**For any further question, contact the investigator**

Name of the investigator Gizaw Hailiye

Phone Number 09 21 49 35 60

*Thank you for consenting to be a participant in this study*.
